# Evaluation of the *ex vivo* Effects of Tamoxifen on Adipose-Derived Stem Cells: A Pilot Study

**DOI:** 10.3389/fcell.2021.555248

**Published:** 2021-03-22

**Authors:** Ilena Boemi, Andrea Vittorio Emanuele Lisa, Eleonora Vitali, Nurçin Liman, Andrea Battistini, Federico Barbera, Luca Maione, Valeriano Vinci, Marco Ettore Attilio Klinger, Andrea Gerardo Antonio Lania

**Affiliations:** ^1^Laboratory of Cellular and Molecular Endocrinology, Humanitas Clinical and Research Center, Istituto di Ricovero e Cura a Carattere Scientifico (IRCCS), Rozzano, Italy; ^2^Department of Medical Biotechnology and Translational Medicine BIOMETRA, University of Milan, Milan, Italy; ^3^Department of Medical Biotechnology and Translational Medicine BIOMETRA, Reconstructive and Aesthetic Plastic Surgery School, University of Milan, Milan, Italy; ^4^Plastic Surgery Unit, Humanitas Clinical and Research Center, Istituto di Ricovero e Cura a Carattere Scientifico (IRCCS), Rozzano, Italy; ^5^Plastic Surgery Unit, Clinica San Carlo, Paderno Dugnano, Italy; ^6^Department of Biomedical Sciences, Humanitas University, Pieve Emanuele, Italy

**Keywords:** adipose-derived stem cells, cell therapies, tamoxifen, autologous fat grafting, breast cancer

## Abstract

Autologous fat grafting (AFG) is a safe and minimally invasive procedure to correct soft tissue defects. The benefit of AFG is attributed to adipose-derived stem cells (ASCs) in fat tissue graft. This technique is useful also in patients undergoing reconstructive surgery following quadrantectomy for breast cancer. However, these patients are frequently treated with tamoxifen. We evaluated the *ex vivo* effects of tamoxifen on ASCs to understand if cellular functions of ASCs are affected. We selected 24 female patients; 10 of which were breast cancer patients treated with quadrantectomy and tamoxifen. As control group, we selected 14 healthy female subjects (9 premenopausal and 5 menopausal). We found that tamoxifen has no effect on cellular proliferation, VEGF secretion or apoptosis of ASCs. The gene expression assessment demonstrated no impairment in differentiation capacity of ASCs. Our results showed that tamoxifen has no effect on cellular functions of ASCs for the first time in an *ex vivo* single-center study.

## Introduction

Autologous fat grafting (AFG), also called fat transplantation or lipofilling, is a safe and minimally invasive surgical procedure to correct soft tissue defects in reconstructive surgery (Pearl et al., 2012). In women, among cancers, the most frequently diagnosed is breast cancer. Breast cancer is also the leading cause of death from cancer in women worldwide (Tao et al., 2015). It is common medical practice to surgically remove primary tumor mass in breast cancer patients and consequently, there is a population of women in need of reconstructive surgery following the tumor excision. So, AFG can be applied first and foremost in breast reconstruction following breast conserving surgery (BCS) of breast cancer patients, providing a remarkable solution to common sequelae of BCS such as post-surgical local deformities and pain syndromes (Caviggioli et al., 2011, 2016; Maione et al., 2014). It can also be used as an alternative therapy in radiotherapy-induced dystrophic tissues (Rigotti et al., 2007), chronic ulcers, scar tissues (Klinger et al., 2015), and degenerative diseases such as systemic sclerosis (Del Papa et al., 2015).

Adipose tissue, commonly known as fat, was initially considered solely as a simple storage organ of excess energy, thermal insulation, and mechanical cushion. It is indeed a metabolically dynamic organ functioning as the primary site of excess energy storage as well as an endocrine organ synthesizing various biologically active compounds. More importantly regarding its application in reconstructive medicine, autologous adipose tissue is biocompatible. The surgical techniques of AFG have changed within the last few years. In the past, it had limited benefits due to the free transfer of intact adipose tissue. More specifically, the technique is based on free composite fat-cell transplantation strategies (Simonacci et al., 2016). Indeed, given the high incidence of breast cancer in women, new techniques for post-surgical breast reconstruction continuously develop, such as the cell-assisted lipotransfer (CAL). CAL consist of transplanting fat with adipose-derived stem cells (Matsumoto et al., 2006). Human adipose-derived stem cells are multipotent mesenchymal stem cells, and they are one of the components of the stromal vascular fraction (SVF) of adipose tissue (Simonacci et al., 2017). The stromal vascular fraction is formed by multiple cell types, including circulating blood cells, fibroblasts, pericytes, endothelial cells, and adipose-derived stem cells (Gir et al., 2012). ASCs, present in the SVF, have been shown to be metabolically active, secreting angiogenic factors such as vascular endothelial growth factor (VEGF) and differentiating into a wide range of cell types in all three embryonic cell lineages. Within the mesodermal lineage, ASCs can differentiate into chondrocytes, myocytes and osteoblasts, whereas only neurogenic differentiation of ASCs has been observed within the ectodermal lineage (Mizuno and Hyakusoku, 2010; Yu et al., 2011; Jung Ho and Yoon, 2019). Current literature also describes hepatic and pancreatic differentiation capacity of ASCs, therefore completing the trigerminal lineage potential (Frese et al., 2016). Consequently, AFG is now a common practice following surgery of breast cancer patients who are frequently under hormone therapy such as tamoxifen prior to surgery (Caviggioli et al., 2011; Maione et al., 2014; Stark et al., 2018).

Tamoxifen (TAM) is a synthetic non-steroidal anti-estrogen that is the hormone therapy of choice in estrogen receptor positive breast cancer pre-menopausal patients (Osborne, 1998). Its anti-estrogenic effect in target tissues (e.g., mammary and adipose tissues) stems from competitive binding to estrogen receptors. Tamoxifen is recommended as a long-term prophylaxis for patients at high risk for developing breast cancer based on reduced recurrence rates (Cuzick et al., 2007). It is also used for the treatment of invasive breast cancer before and/or after surgery, for preventing invasive breast cancer in women at high risk for developing breast cancer, and lastly for post-operative medical management of ductal carcinoma *in situ* (DCIS) (Early Breast Cancer Trialists’ Collaborative Group [EBCTCG], 2011).

However, in 2015 Pike et al. concluded that *in vitro* exposure to tamoxifen has cytotoxic effects on ASCs in terms of increased apoptosis and inhibition of proliferation as well as reduction of multipotent differentiation capacity in a dose and time dependent manner (Pike et al., 2015). A systematic review of patient factors affecting ASC viability indicated a reduction of proliferation and differentiation potential of ASCs with increasing age, body mass index, diabetes mellitus and exposure to radiotherapy and tamoxifen; although, the latter was not uniformly seen across all studies (Varghese et al., 2017). Moreover, in some human and animal models tamoxifen has been shown to inhibit proliferation of endothelial cell lines and decrease VEGF production (Blackwell et al., 2000; McNamara et al., 2001). On the other hand, concerning the unpredictable and relatively high rates of fat graft resorption in AFG, recent clinical observations suggest that tamoxifen exposure reduces resorption and fibrosis rates of injected fat grafts, resulting in a better integration of autologous fat grafts (Silva et al., 2018).

AFG following hormone therapy in breast cancer patients is increasingly used, however in literature there is not yet a clear understanding about this procedure in TAM treated patients. The aim of this study is to investigate the *ex vivo* effects of tamoxifen on ASCs obtained from ER-positive breast cancer patients and to compare the cellular functions of ASCs obtained from TAM treated patients with that of control group subjects. In further detail, ASCs extracted from SVF of liposuctioned adipose tissue of breast cancer patients previously treated with tamoxifen have been examined in terms of proliferation, apoptosis, VEGF secretion and multipotent differentiation capacity, and compared with that of control group subjects. Our results showed that tamoxifen has no effect on cellular functions of adipose-derived stem cells and suggested that even in patients with breast cancer, treated with tamoxifen, AFG can be remarkable solution to common sequelae of BCS.

## Materials and Methods

### Patients Selection

The study was approved by the Ethical Committee of Humanitas Research Hospital (study number: 1960, ID of the experimentation: 545, authorization date: 08-03-2018). Informed consent was obtained from all patients involved in the study. The research was carried out according to The Code of Ethics of the World Medical Association (Declaration of Helsinki).

For this study we selected a total of 24 female patients; 10 of which were pre-menopausal ER positive breast cancer patients treated with tamoxifen (20 mg per day for at least 6 months). As the control group, we selected 14 healthy females, 9 pre-menopausal and 5 menopausal subjects. The age of our patients ranged between 18 and 64 years and the mean age is of 42 years. In detail, the subjects enrolled in the study were divided into three groups. The first group was composed of women in daily treatment with tamoxifen (for at least 6 months) for oncological reasons suffering from post-mastectomy pain syndrome and needing autologous fat grafting. The tamoxifen dose was fixed at 20 mg/day and we included all the patients that were taken the drug with the fixed protocol. The second group was composed of fertile women presenting with retractile scars requiring autologous fat grafting (hormonal status was confirmed by FSH and LH blood test, subjects assuming hormones were excluded). The third group was composed of post-menopausal women (at least 1 year from the last menstruation) treated with autologous fat grafting for aesthetic purpose. All tamoxifen treated subjects included in the study were compliant. In all the subjects enrolled in the study there were no co-morbidity, and they were not receiving other concomitant therapies. The exclusion criteria included: tobacco, pregnancy, body mass index (BMI) >25 or <19, and any other comorbidity. For each sample, fat tissue was drawn bilaterally from the flanks. Infiltration solution was made of saline and adrenaline (1 cc in 500 mL). No local anesthetic was used during the procedure. Procession is obtained through Coleman technique (centrifugation 3,000 rpm for 3 min). The adipose tissue of breast cancer patients was obtained while they were still continuing the tamoxifen therapy. The patients would take the treatment early in the morning and the adipose tissue collection was performed later in the day. Subjects enrolled in the study were not treated with antithrombotic DVT prophylaxis. Supplementary Table S1 describes, in detail, participant information.

### Isolation and Cell Culture

Adipose-derived stem cells (ASCs) were obtained from side lipoaspiration. Liposuctioned adipose tissue was washed with PBS (Sigma-Aldrich) and then digested with collagenase I (1 mg/ml) (Merck Millipore) for 1 h at 37°. After tissue centrifugation at 1,200 g for 10 min, cells were resuspended in 160 mM NH_4_Cl to remove erythrocytes. ASCs were then cultured in phenol red free DMEM medium (Gibco) and supplemented with 10% FBS (Sigma-Aldrich), 100 U/ml penicillin (Lonza), 100 μg/ml streptomycin (Lonza) and 1 mM L-glutamine (Lonza). The cells were submitted for the experiment at passages 2–6.

HUVEC cells were kindly provided by Dr. Elisa Zaghi (Humanitas Clinical and Research Hospital, Rozzano, Italy). Cells were grown on plates coated with collagen (50 μg/ml) in EGM2 medium (Lonza) supplemented with 5% FBS. HUVECs that were ∼80% confluent were used for experiment.

### Immunophenotype

Immunophenotype of ASCs was analyzed between passage 1–2 of culture. The cells were stained with the following fluorescent-conjugated antibodies: CD44FITC, CD90(Thy1)PE/Cy5, CD105PE/Cy7, CD73(Ecto-5′-nucleotidase)PerCP/Cy5.5, CD45PB, CD34APC/Cy7. All the antibodies were purchased from Biolegend. The samples were acquired using FACS SYMPHONY (BD Biosciences) and the results were analyzed using FACSDiva software (BD Biosciences). The gating strategy and the representative histograms of ASCs expression marker are shown in Supplementary Figure S1.

### Proliferation Assay

Cells were plated in 96-well plates (15 × 10^3^ cells/well) and after 4 and 8 days of culture cell proliferation was analyzed using the CyQUANT Cell Proliferation Assay Kit (Invitrogen) according to the manufacturer’s protocol. The fluorescent intensity was read in a microplate reader with the appropriate filters.

### Apoptosis Assay

Detection of apoptosis was performed using FITC Annexin V Apoptosis Detection Kit with 7-AAD (#640922, Biolegend), according to the manufacturer’s protocol. After 48 h of culture cells were double-stained with the Fluorochrome-labeled Annexin V and the fluorescent 7-AAD dye for 15 min at room temperature in the dark. The percentage of apoptotic cells was determined by flow cytometry using FACS SYMPHONY (BD Biosciences). The results were analyzed using FACSDiva software (BD Biosciences).

### Multilineage Differentiation

For adipogenic differentiation, ASCs were cultured in adipogenic induction medium containing phenol red free DMEM medium (Gibco), supplemented with 10% FBS (Sigma-Aldrich), 100 U/ml penicillin (Lonza), 100 μg/ml streptomycin (Lonza), 1 mM L-glutamine (Lonza), 1 μM dexamethasone, 500 nM IBMX, 50 μM indomethacin and 10 μg/mL human insulin for 21 days. After 3 weeks cells were fixed and stained with fresh Oil Red-O solution (Sigma-Aldrich) to identify lipid droplets.

For osteogenic differentiation, ASCs were cultured in osteogenic induction medium containing phenol red free DMEM medium (Gibco), supplemented with 10% FBS (Sigma-Aldrich), 100 U/ml penicillin (Lonza), 100 μg/ml streptomycin (Lonza), 1 mM L-glutamine (Lonza), 50 μM ascorbic acid, 100 nM dexamethasone and 10 mM β-glycerophosphate for 21 days. After 3 weeks cell fixed and stained with Alizarin Red Staining Solution (Merck) to identify calcium deposits.

Images acquired with EVOS XL Imaging System (LifeTecnologies).

### VEGF Secretion and Expression

To analyze VEGF-A secretion, ASCs were cultured for 4 and 8 days. At each time point the collected supernatants were used to measured human VEGF-A secretion with ELISA kit (Duo-Set ELISA, R&D Systems), according to manufacturer protocol. The absorbance was measured in a microplate reader at 450 nm. The experiment was performed in triplicate for each sample. To analyzed VEGF-A gene expression, ASC were cultured for 24 h and then harvested for RNA extraction. As controls we used HUVEC cells untreated or treated with 10 nM phorbol-12-myristate-13-acetate (PMA) (Sigma-Aldrich) for 24 h. Then HUVECs were harvested for RNA extraction.

### Quantitative RT-PCR Analysis

Total RNA (200 ng) was extracted from cultured ASCs, for each sample, and HUVECs with SV Total RNA Isolation System (Promega) and converted to cDNA using GoTaq(R) 2-Step RT-qPCR System (Promega) according to the manufacturer protocol. mRNA levels were quantified with Viia7 (Applied Biosystems) using SYBR Green Real-Time PCR [GoTaq(R) 2-Step RT-qPCR System, Promega] according to manufacturer protocol. Glyceraldehyde 3-phosphate dehydrogenase (GAPDH) was used as internal control. Primers used to detect the specific genes are detailed in Supplementary Table S2.

### Statistical Analysis

GraphPad Prism 7.0 was used to perform statistical analyses. Comparison among multiple groups were done with ANOVA test and *post hoc* Bonferroni’s correction, whereas comparisons between two groups were done with unpaired *t*-test. A value of *p* < 0.05 was considered statistically significant. All data are shown as mean ± S.E.M.

## Results

### Tamoxifen Did Not Affect Proliferation of ASCs

**FIGURE 1:**
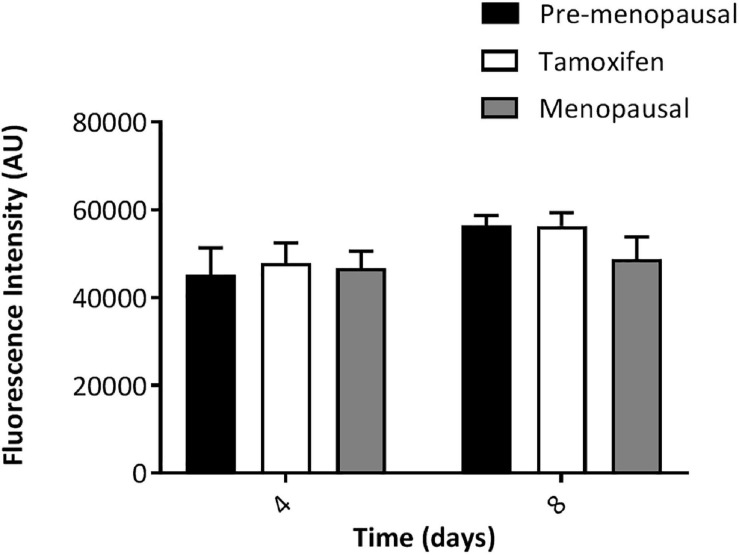
*Ex vivo* effect of tamoxifen on ASCs proliferation. ASCs were left in culture for 4 or 8 days and then proliferation rate was evaluated. Data represent mean ± S.E.M. of three independent experiments each performed in triplicates.

In order to evaluate the *ex vivo* effects of tamoxifen we firstly assessed the ability to proliferate of ASCs. Prior to submit the cells for experiments, between passage 1–2, ASCs were checked for immunophenotype at FACS SYMPHONY (BD Biosciences) (Supplementary Figure S1). As shown in Figure 1. we evaluated cell proliferation at two different time point, day 4 and day 8 of culture, and we observed no difference between ASCs from pre- and menopausal patients compared to tamoxifen treated ones. This data suggest that tamoxifen didn’t affect cell proliferation of *ex vivo* ASCs.

### Tamoxifen Did Not Increase Apoptosis

**FIGURE 2:**
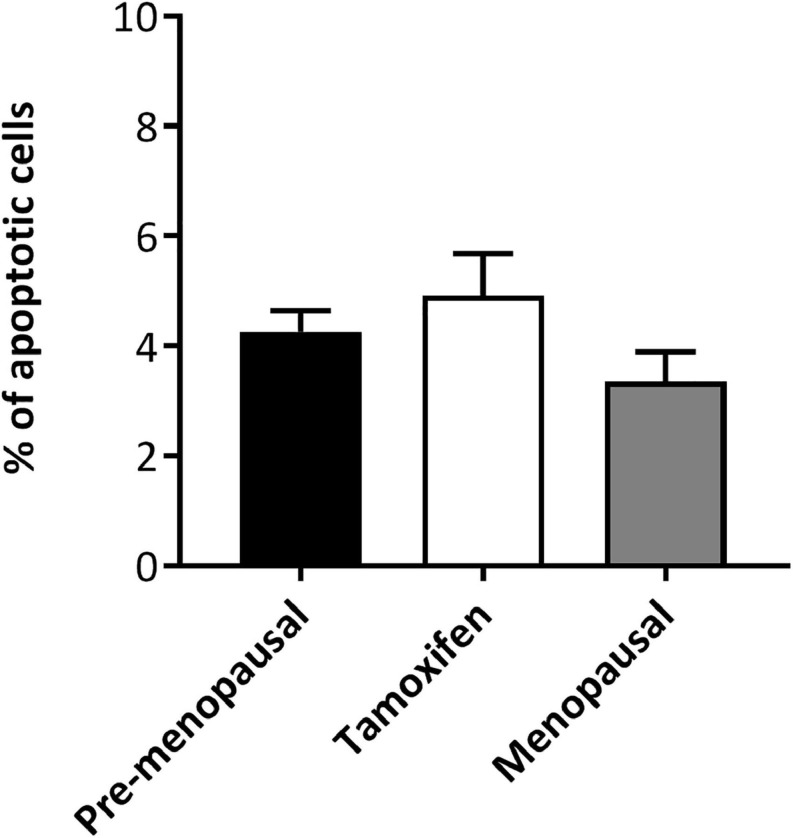
*Ex vivo* effect of tamoxifen on apoptosis of ASCs. TAM did not significantly increased apoptosis of ASCs. Cells were plated for 48 h and then apoptosis was analyzed by flow cytometry, apoptotic cells were defined as Annexin V + 7-AAD-. Data represent mean ± S.E.M. of three independent experiments.

To investigate the effect of tamoxifen on apoptosis of ASCs, cells from pre-, menopausal and tamoxifen treated patients were plated for 48 h and then apoptosis was analyzed by flow cytometry. As shown in Figure 2, tamoxifen did not increase the apoptosis of ASCs from treated patients compared to ASCs of control ones.

### Effects of Tamoxifen on VEGF

**FIGURE 3:**
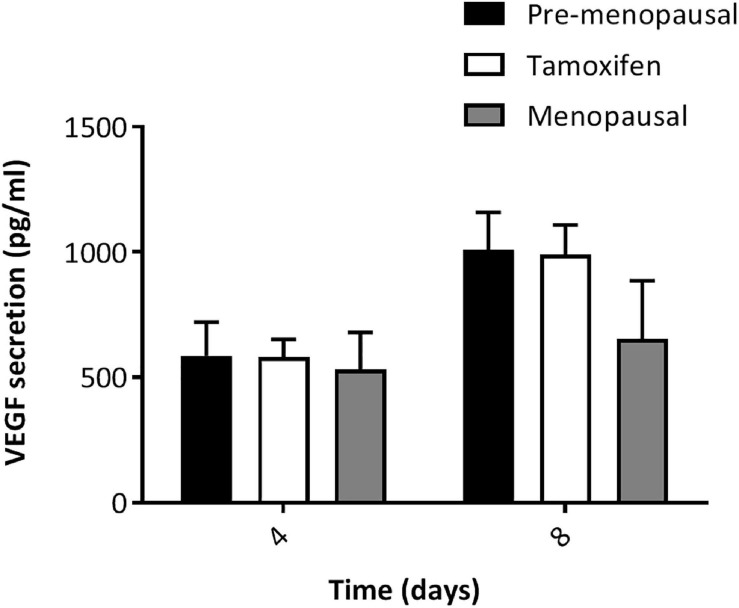
VEGF-A secretion by ASCs. ELISA analysis of VEGF released (pg/ml) by ASCs at 4 and 8 days of culture. ASCs from TAM treated patients did not display lower secretion and at day 8 they were able to secret a higher amount of VEGF compared to day 4; same behavior observed as for ASCs from pre- and post- menopausal patients. Data represent mean ± S.E.M. of three independent experiments each performed in triplicates.

We wanted to analyze the ability of ASCs from TAM-treated patients to secrete VEGF-A. ASCs form pre-, menopausal and tamoxifen patients were cultured for 4 and 8 days, then VEGF secretion was assessed on supernatant. As shown in Figure 3, there was no difference in the secretion of VEGF by tamoxifen group compare to control group, moreover ASCs from TAM-treated patients displayed the same trend of control ones. To confirm our data, we also performed qRT-PCR for the expression of VEGF-A (Supplementary Figure S2) and we used as controls HUVEC cells and HUVEC cells treated for 24 h with PMA, that is known from the literature to increase VEGF expression. As shown by the real time analyses, there was no differences in the expression of VEGF by tamoxifen group compare to control group. Taken together our results indicate that tamoxifen didn’t affect VEGF secretion and expression.

### Effects of Tamoxifen on Multilineage Differentiation

**FIGURE 4:**
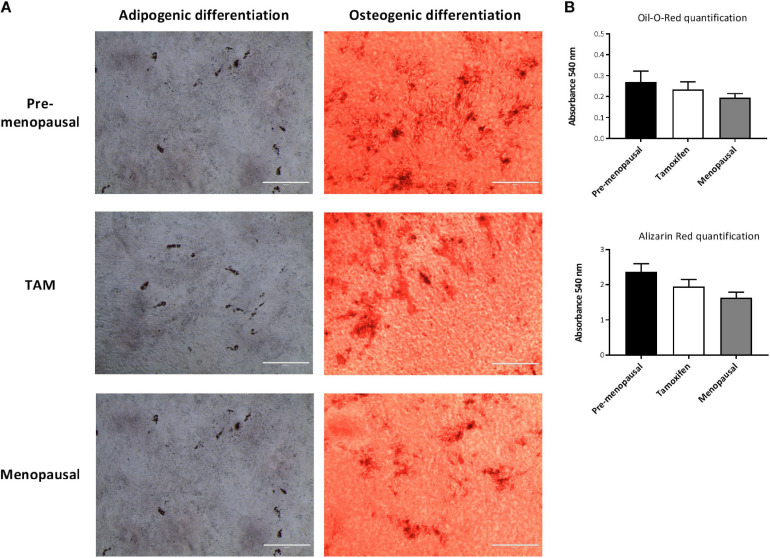
Effects of tamoxifen on multilineage differentiation. ASCs were cultured for 3 weeks with appropriated induction media for adipogenic or osteogenic differentiation to assess the multilineage differentiation capabilities. **(A)** Representative images of Oil Red O (left panels) and Alizarin Red (right panels) staining of ASCs. Oli Red O stains intracellular lipids, while Alizarin Red stains calcified extracellular matrix. Scale bar: 400 μm. **(B)** Quantification of Oil Red O and Alizarin Red staining of ASCs shown no differences in multilineage differentiation capabilities between the three groups. Data represent mean ± S.E.M.

We assessed the effects of tamoxifen on multilineage differentiation of ASCs after 21 days. As shown in Figure 4A ASC from pre-, post- menopausal and TAM treated patients exhibit capacity for osteogenic and adipogenic differentiation, when cultured with the specific induction media, and stained with Alizarin Red and Oil-O-Red, respectively. The quantification of Alizarin Red and Oil-O-Red staining highlight no differences in the ability of differentiate between the 3 groups (Figure 4B).

**FIGURE 5:**
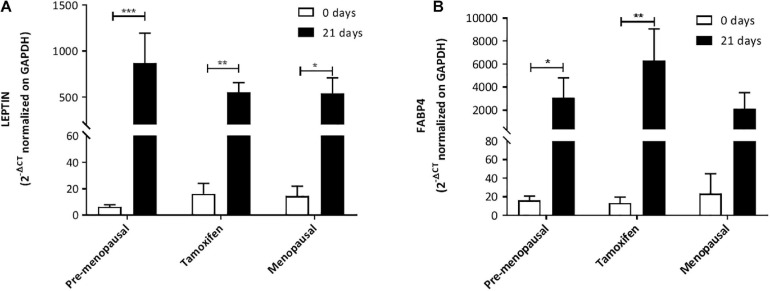
Expression of adipogenic markers. Expression of adipogenic markers LEP **(A)** and FABP4 **(B)** mRNA levels from qRT-PCR analysis highlight that there is no significant alteration between the three groups. Moreover, the analysis confirmed that ASCs from all three groups are able to increase the expression of LEP and FABP4 after 21 days of culture with the induction media compared to day 0. Data in all graphs are presented as mean ± S.E.M. *p < 0.05, **p < 0.01, and ***p < 0.001.

We performed also quantitative real-time RT-PCR for osteogenic and adipogenic marker, OST (Osteocalcin), ALP (Alkaline Phosphatase) and LEPTIN, FABP4 (Fatty Acid-Binding Protein 4) respectively. No significant alterations were founded in the expression of adipogenic marker between the 3 groups and moreover all ASC extracted from pre-, post- menopausal and TAM treated patients upregulate LEPTIN and FAPB4 after 21 days with the induction media compared to day 0, indicated that ASCs were able to differentiate into adipocytes (Figures 5A,B).

**FIGURE 6:**
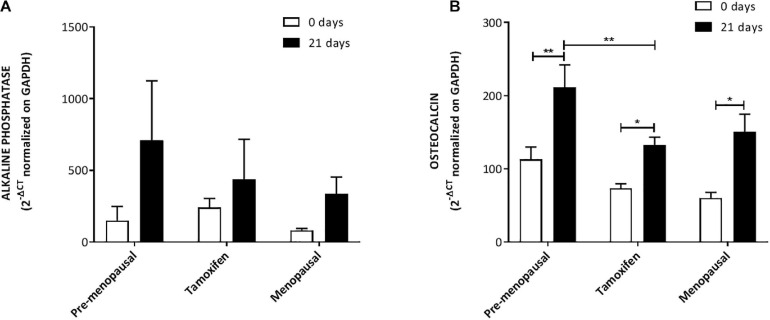
Expression of osteogenic markers. Expression of osteogenic markers OST **(A)** and ALP **(B)** mRNA levels from qRT-PCR analysis. the analysis confirmed that ASCs from all three groups are able to increase the expression of ALP and OST after 21 days of culture with the induction media compared to day 0. Data in all graphs are presented as mean ± S.E.M. *p < 0.05 and **p < 0.01.

We assed also the osteogenic potential and as highlighted by qRT-PCR analysis; no differences were observed in ALP expression level and all the ASC had a higher expression at day 21 compared to day 0 (Figure 6A). OST analysis reveals that ASCs from TAM treated patients had a lower expression of this gene compared to pre-menopausal ASCs but not to post-menopausal ones. Despite this ASC extracted from pre, post- menopausal and TAM treated patients upregulate OST after 21 days with the induction media compared to day 0 (Figure 6B).

### ASCs Expression of ERα and ERβ

**FIGURE 7:**
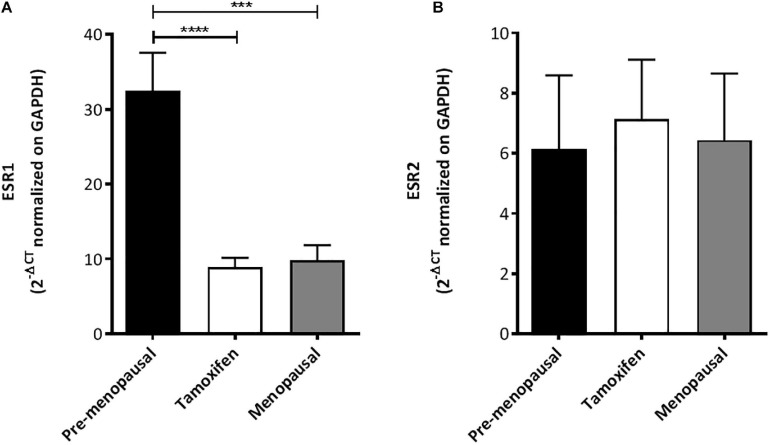
Expression of estrogen receptor by ASCs. **(A)** qRT-PCR analysis for ESR1 (ER-α) shown that ASCs from pre-menopausal patients have a significantly high level of ER-α compare to the others two groups. **(B)** No differences were found in ESR2 (ER-β) mRNA levels within the three group as shown by qRT-PCR analysis. ***p < 0.001 and ****p < 0.0001.

ER presences in ASCs were evaluated using qRT-PCR. As shown in Figures 7A,B, both ER-α (ESR1) and ER-β (ESR2) were detected in human ASCs. Quantitative RT-PCR analysis reveals that ER-α levels in ASCs were higher than ER-β; moreover, despite no differences within the 3 groups in ER-β levels we observed that ASCs from pre-menopausal patients have a significantly high level of ER-α compare to the others two groups. No differences were observed in the levels of ER-α between ASC from TAM treated patients and post-menopausal one.

## Discussion

This study evaluated the *ex vivo* effects of tamoxifen treatment on adipose-derived stem cells. It aims to provide a further understanding of the impact of endocrine therapy adjuvant to surgical approaches in breast cancer patients, and more specifically of selective estrogen receptor modulators such as tamoxifen, on adipose-derived stem cells present in autologous fat grafts used for breast reconstruction. Our findings suggest that tamoxifen has no significant effect on the cellular functions of adipose-derived stem cells, justifying autologous fat grafting as the standard treatment for breast reconstruction in post-surgical breast cancer patients treated with tamoxifen.

Given the anti-apoptotic, angiogenic and anti-oxidant features of adipose-derived stem cells, autologous fat grafting enriched with adipose-derived stem cells has become the standard surgical practice (Serra-Renom et al., 2010; Sterodimas et al., 2010; Nie et al., 2011). Autologous fat grafting is a safe and minimally invasive surgical procedure to correct soft tissue defects in the field of reconstructive surgery (Pearl et al., 2012). The latter procedure is frequently performed in breast cancer patients after surgical removal of the mammary tumor mass (Caviggioli et al., 2011; Maione et al., 2014; Stark et al., 2018). It comprises of obtaining a fat graft from a donor site through liposuction and re-injecting it immediately into the region of interest through syringes. For more successful graft survival, these fat grafts can be augmented with adipose-derived stem cells. Most laboratories digest the lipoaspirate with collagenase following centrifugation to generate the stromal vascular fraction, which consists of multiple cell types including adipose-derived stem cells (Gir et al., 2012). However, most of these patients are treated with adjuvant endocrine therapy, tamoxifen being the first choice in premenopausal women (Osborne, 1998). In consideration of this concomitance, it is necessary to study the effects of tamoxifen on adipose-derived stem cells, present in the SVF, to further investigate its impact on autologous fat grafts used in breast reconstruction.

Tamoxifen is a non-steroidal selective estrogen receptor modulator (SERM) that inhibits the growth of breast cancer cells by competitive antagonism of the estrogen receptor (ER) (Osborne, 1998). The current literature on the impact of tamoxifen on adipose-derived stem cells commonly used to augment autologous fat grafts is limited. Pike and colleagues studied the impact of tamoxifen on adipose-derived stem cells in *in vitro* conditions and demonstrated that tamoxifen (at a concentration above 2 μM) has cytotoxic effects on adipose-derived stem cells in terms of apoptosis induction and proliferation inhibition. However, the cell cycle in adipose-derived stem cells was unchanged. In the same study, tamoxifen also appears to decrease the multipotent differentiation capacity of adipose-derived stem cells (Pike et al., 2015). These findings are in line with previous work in which it was shown that high concentrations of tamoxifen induce caspase dependent apoptosis (Mandlekar and Kong, 2001). Furthermore, *in vitro* cells are exposed to an entirely non-metabolized pharmacological compound, whereas *in vivo* cells are in contact with a pool of metabolites of the parent drug at diverse concentrations in relation to bioavailability and distribution. In this respect, the study conducted by Lien et al. showed how tamoxifen and its active metabolites are distributed in tissues from rat and human. In the rat, tamoxifen was present with the highest concentration in the lung and liver. In the other tissues, researchers found that the levels of TAM and its metabolites were 8- to 70-fold higher than in serum. Concentration fluctuations of tamoxifen and its metabolites were observed during one dosing interval in most tissues, except fat and testes, in which the drug concentrations were relatively stable. In the adipose tissue TAM active metabolites were present just in a tiny amount. Experiments were conducted also in human healthy and malignant tissues and the concentration of TAM and its active metabolites, present in these tissues, confirmed the conclusions drawn in the aforementioned experiments on rats. The concentration of tamoxifen was 10- to 60-fold higher in human tissues compared to serum, and, as seen in the rat, TAM was present with the highest level in the lung and liver. The greater quantity of the drug was retained by pancreas, pancreatic tumor, brain metastases from breast cancer and primary breast cancer. Concerning the active metabolites, their concentration was higher in most tissues, except fat.

Adipose tissue contained high concentration of the parent drug and low concentrations of its active metabolites (Lien et al., 1991). Our studies suggest that tamoxifen treatment does not significantly affect the proliferation capacity and apoptosis rates of *ex vivo* adipose-derived stem cells of patients subjected to prolonged treatment in comparison to that of control group composed of both pre-menopausal and menopausal women.

Angiogenesis is an important component in the fat graft survival due to the necessity of vascular supply to the newly implanted tissues (Nishimura et al., 2000). Volume reduction followed by failure of autologous fat grafts is an increasingly incident event in reconstructive surgeries. A novel technique to overcome this issue is augmenting the autologous fat grafts with adipose-derived stem cells innately present in the SVF of adipose tissues. The better preservation of fat tissue architecture in autologous fat grafts enriched with adipose-derived stem cells (ASCs) is mainly attributed to the angiogenic properties of ASCs (Gentile et al., 2012). However, one of the target population for this procedure, namely breast cancer patients, are commonly treated with tamoxifen prior to autologous fat grafting. For this reason, it is imperative to investigate if tamoxifen has any influence on the angiogenesis of adipose-derived stem cells. In our study the angiogenesis of ASCs is quantitated in terms of vascular endothelial growth factor (VEGF-A) secretion and expression by the adipose-derived stem cells. Regarding the role of VEGF in wound healing, a study showed that during wound repair the expression of VEGF and its receptor increased, indicating a significant role for this mediator in wound angiogenesis (Frank et al., 1995). Besides angiogenesis, another study concluded that VEGF stimulates wound healing via multiple mechanisms including collagen deposition and epithelization (Bao et al., 2009). Therefore, VEGF secretion by adipose-derived stem cells is essential for its angiogenic features utilized in the autologous fat grafting. In this manner, analysis of VEGF-A secretion by the adipose-derived stem cells enable us to indirectly measure the angiogenic capabilities of our cells. In our studies we found that there was no significant difference in the secretion and expression of VEGF-A by the ASCs of the group of tamoxifen treated patients compared to that of the control group. Moreover, ASCs of tamoxifen treated patients displayed the same trend as that of control group; in both groups, VEGF secretion increased with longer duration of culture. Based on these two observations, it can be suggested that tamoxifen did not affect VEGF-A secretion and expression by adipose-derived stem cells.

Human adipose-derived stem cells (ASCs), originating from the vascular stromal compartment of adipose tissue, are multipotent cells able to differentiate into several cells types including osteoblasts, chondrocytes and adipocytes (Lindroos et al., 2011). Such multipotency and easy accessibility of adipose-derived stem cells make it a suitable candidate for use in many diverse regenerative therapies for damaged bones, myocardial tissues, chronic wounds and many others (Gimble et al., 2007). One such application of ASCs in reconstructive surgery is the autologous fat grafting augmented with adipose-derived stem cells for breast reconstruction in women with breast cancer. However, there are significant confounders including adjuvant endocrine therapy such as tamoxifen, in evaluating the success rates of such procedures this population of patients. Given the complex and cytotoxic nature of the therapeutic regimens for most cancer patients, the efficacy of ASC augmentation has been questioned by many practitioners. According to previous reports, bone marrow derived mesenchymal stem cells are resistant to chemotherapeutic agents and irradiation (Chen et al., 2006). Nevertheless, there is limited and contradicting literature on the effects of cytotoxic therapies on adipose-derived stem cells. A recent study outlined that ASCs are resistant to drugs widely used for chemotherapy, such as cisplatin, comptothecin, and vincristine, and that they can preserve their phenotype and the ability to differentiate *in vitro* after treatment (Liang et al., 2011). In contrast, another study showed that the capacity for multilineage differentiation was down-regulated in ASCs exposed to 5μM of tamoxifen (Pike et al., 2015). In light of these conflicting results, we studied the multilineage differentiation capacity of the adipose-derived stem cells obtained from our control group and tamoxifen-treated patients. We compared the ability to differentiate at day 0 and after 21 days of culture with the induction medium. As shown in the graphs, all groups are able to induce the gene after 21 days of culture, and this induction, with the exception of ALP gene, is statistically significant compare to day 0. The results of the real time for LEP and ALP gene shown a non-statistically significant trend comparing TAM treated patients and pre-menopausal subjects. It is interesting to note that TAM treated patients are mostly similar to post-menopausal subjects, in terms of differentiation capacity, compare to pre-menopausal ones. A recent study shown that at a transcriptome level ASCs from pre- e post- menopausal women are different (Xie et al., 2020), this could, at least partially, explaining the trend and why TAM-treated ASCs and ASCs from post-menopausal subjects appear to be more similar and slightly different from pre-menopausal one. At the best our knowledge our study is the first implying *ex vivo* ASCs from TAM-treated patients and pre- and post-menopausal subjects, indeed in light of the trend we found and the above-mentioned study, further studies to better clarify this point are needed. Overall, in our study, there is no significant difference in terms of multilineage differentiation capacity among all of the ASCs obtained from pre-menopausal subjects, menopausal subjects and tamoxifen treated patients. This finding is in parallel with the literature on bone marrow derived mesenchymal stem cells as well as the report on cisplatin, comptothecin, and vincristine (Frank et al., 1995; Chen et al., 2006).

Estrogen is an endogenous hormone essential for growth of various types of cells including those in mammary and adipose tissues. Estrogen exerts these effects through two nuclear estrogen receptors, namely ERα and ERβ (Molina et al., 2017). Estrogen receptor expression at the time of diagnosis of breast cancer is an invaluable prognostic factor also used for personalizing targeted therapies (Thomas and Gustafsson, 2011). The mechanism of action of one of the adjuvant endocrine therapy, namely selective estrogen receptor modulators such as tamoxifen, involves these estrogen receptors expressed in the breast cancer cells. The estrogen receptor antagonism exerted by tamoxifen inhibits the growth signal in breast cancer and so, the initially estrogen dependent tumor mass undergoes regression upon deprivation of their supporting hormone. Endocrine treatment, concurrently or not with chemotherapy, has been shown to reduce the recurrence and mortality rates in breast cancer positive for ER (Early Breast Cancer Trialists’ Collaborative Group [EBCTCG], 2018). However, estrogen receptor expression is considerably ubiquitous and indeed, the effect of tamoxifen is beyond the scope of breast cancer cells only (Hua et al., 2018). Concerning the effects of estrogen receptor activation in ASCs, the available scientific literature is limited. A recent study found that adults stem cells from adipose tissues expressed both ERα and ERβ at the mRNA level and ERβ at the protein level (Pike et al., 2015). Another study, performed on ASC from mouse, concluded that both ERα and ERβ act as positive regulators improving ASC proliferation, migration and wound healing (Zhang et al., 2016). Likewise, our study involved a preliminary evaluation of the expression of estrogen receptors in our adipose-derived stem cells. Both of ERα and ERβ were detected in the human adipose-derived stem cells with ERα levels higher than that of ERβ. This finding is in parallel with previous studies concluding ERα to be the main mediator of proliferation, wound healing and migration in mouse adipose-derived stem cells (Zhang et al., 2016). Although no significant differences in ERβ levels were observed among the three groups, the ASCs obtained from pre-menopausal subjects had a significantly higher level of ERα expression compared to that of other two groups with equivalent levels. Nevertheless, the roles of ERα and ERβ in adipose-derived stem cell biology will require further elucidation.

Breast reconstruction following breast cancer surgery is well known to improve quality of life. Autologous fat grafting has become a standard of care in the repair of volume and contour defects, and in improving unaesthetic results such as indurations, scar contractures, tissue deficits and breast asymmetry. In spite of the overall positive patient and surgeon reported outcomes, the concomitant administration of long-term adjuvant endocrine therapy confounds the success rate analysis of autologous fat grafting in breast cancer populations (Liang et al., 2011; Millet et al., 2016; Lindegren et al., 2019). Our work focused on the *ex vivo* effects of tamoxifen, another antiestrogen therapy, on cellular functions of adipose-derived stem cells. In this study we shown, for the first time in an *ex vivo* single-center study, that tamoxifen does not impair the cellular functions of ASCs. For this reason, we hypothesize that tamoxifen treatment, post-mastectomy, should not contraindicate autologous fat grafting in breast cancer patients.

## Data Availability Statement

The original contributions presented in the study are included in the article/Supplementary Material, further inquiries can be directed to the corresponding author/s.

## Ethics Statement

The studies involving human participants were reviewed and approved by the Ethical Committee of Humanitas Research Hospital (study number: 1960, ID of the experimentation: 545, authorization date: 08-03-2018). The patients/participants provided their written informed consent to participate in this study.

## Author Contributions

IB, AVL, MK, and AGL: conceptualization. IB: methodology and formal analysis. IB, AVL, EV, and NL: validation. IB and AVL: investigation. IB, AVL, MK, AGL, EV, AB, and VV: resources. IB, AVL, FB, LM, and VV: data curation. IB, AVL, and NL: writing—original draft preparation. IB, AVL, NL, EV, MK, and AGL: writing—review and editing. IB, AVL, AB, and LM: visualization. MK and AGL: supervision, project administration, and funding acquisition. All authors have read and agreed to the published version of the manuscript.

## Conflict of Interest

Since 1st December 2020, IB has been employed by Frontiers Media SA. IB declared his/her affiliation with Frontiers, and the handling editor states that the process nevertheless met the standards of a fair and objective review. The remaining authors declare that the research was conducted in the absence of any commercial or financial relationships that could be construed as a potential conflict of interest.
