# Supplementary material for: Evaluation of the ex vivo Effects of Tamoxifen on Adipose-Derived Stem Cells: A Pilot Study
Source: Front Cell Dev Biol. 2021 Mar 22;9:555248. doi: 10.3389/fcell.2021.555248 (PMC8019789; doi:10.3389/fcell.2021.555248)
Supplement: Supplementary file 1 [file Image_1.pdf]

Supplementary Material

1.1 Supplementary Figures

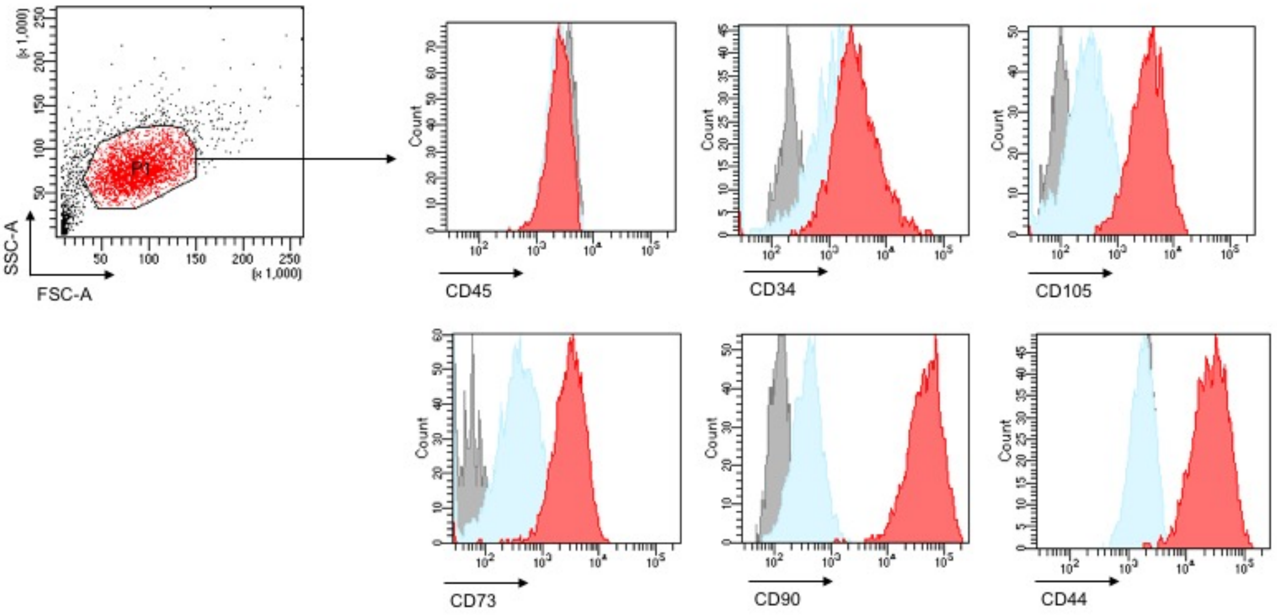

Supplementary Figure 1. Representative gating strategy and representative histograms for assessing ASC phenotype. The ASCs were cultured in phenol red free DMEM medium supplemented with 10% FBS and between passage 1-2 the expression of the following surface antigens was determined by flow cytometry: CD45, CD34, CD105, CD73, CD90 and CD44. Unstained ASC (grey histogram) and FMO (fluorescent minus one, light-blue histogram) ASCs serving as a negative control. Cells were first gated based on morphological appearance, then doublet were excluded, and viable cells were selected to assess the expression of the chosen markers.
