# Supplementary material for: Evaluation of the ex vivo Effects of Tamoxifen on Adipose-Derived Stem Cells: A Pilot Study
Source: Front Cell Dev Biol. 2021 Mar 22;9:555248. doi: 10.3389/fcell.2021.555248 (PMC8019789; doi:10.3389/fcell.2021.555248)
Supplement: Supplementary file 2 [file Image_2.pdf]

Supplementary Material

1.1 Supplementary Figures

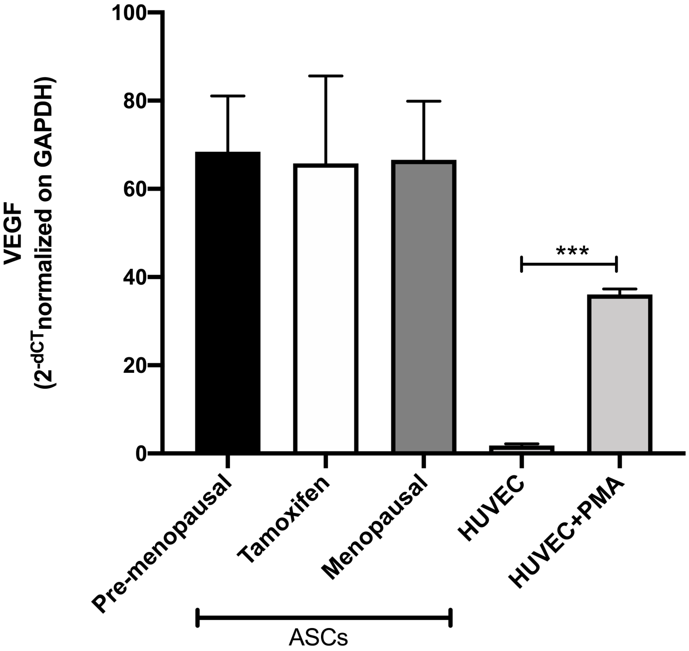

Supplementary Figure 2. Expression of VEGF-A by ASCs and HUVECs. qRT-PCR analysis shown no differences in VEGF mRNA levels between TAM treated group and control ones. HUVEC cells were used as control. After 24h of culture untreated cells expressed low mRNA level of VEGF, and cells PMA treatment (10 nM for 24 Hours) induced a significant increase in VEGF expression (*** $p \leqq 0.001$).
