# Supplementary material for: Evaluation of the ex vivo Effects of Tamoxifen on Adipose-Derived Stem Cells: A Pilot Study
Source: Front Cell Dev Biol. 2021 Mar 22;9:555248. doi: 10.3389/fcell.2021.555248 (PMC8019789; doi:10.3389/fcell.2021.555248)
Supplement: Supplementary file 3 [file Table_1.DOCX]

Supplementary Material

***Table S1.*** Clinical information of 24 participants (LH-RH: luteinizing hormone-releasing hormone analogue; FSH: follicle-stimulating hormone; LH: luteinizing hormone).
