# Supplementary material for: Evaluation of the ex vivo Effects of Tamoxifen on Adipose-Derived Stem Cells: A Pilot Study
Source: Front Cell Dev Biol. 2021 Mar 22;9:555248. doi: 10.3389/fcell.2021.555248 (PMC8019789; doi:10.3389/fcell.2021.555248)
Supplement: Supplementary file 4 [file Table_2.DOCX]

Supplementary Material

**Table S2.** Primers used to detect specific genes.

| **Gene Name** | **Sequence (5’-3’)** | **Producer** |
| --- | --- | --- |
| H_ESR1 FW | GATGAAAGGTGGGATACGAAAAGA | Sigma-Aldrich |
| H_ESR1 RW | CTCTGGCGCTTGTGTTTCAA | Sigma-Aldrich |
| H_ESR2 FW | Predesigned SYBER Green Primers | Sigma-Aldrich |
| H_ESR2 RW | Predesigned SYBER Green Primers | Sigma-Aldrich |
| H_Leptin FW | TCACCAGGATCAATGACATTTCA | Sigma-Aldrich |
| H_Leptin RW | CCAAACCGGTGACTTTCTGTTT | Sigma-Aldrich |
| H_FABP4 FW | TTGACGAAGTCACTGCAGATGA | Sigma-Aldrich |
| H_FABP4 RW | CAGGACACCCCCATCTAAGGT | Sigma-Aldrich |
| H_OSTEOCALCIN FW | GGCTCCCAGCCATTGATACA | Sigma-Aldrich |
| H_OSTEOCALCIN RW | CAGGAGGGCAGCGAGGTA | Sigma-Aldrich |
| H_ALKALINE PHOSPHATASE FW | GATGGCAGTGAAGGCTTCTT | Sigma-Aldrich |
| H_ALKALINE PHOSPHATASE RW | CCGTGGCAACTCTATCTTTGG | Sigma-Aldrich |
| H_GAPDH FW | GATCATCAGCAATGCCTCCT | Sigma-Aldrich |
| H_GAPDH RW | TGTGGTCATGAGTCCTTCCA | Sigma-Aldrich |
